# Combined heat and exercise stress disrupt gut microbiota and promote microbial translocation

**DOI:** 10.3389/fmicb.2026.1779295

**Published:** 2026-05-25

**Authors:** Leizi Min, Alimjan Ablitip, Qingyuan Wang, Ting Li, Qian Di, Xindong Ma, Wen Fang

**Affiliations:** 1 School of Sports Science, East China University of Science and Technology, Shanghai, China; 2 Division of Sports Science & Physical Education, Tsinghua University, Beijing, China; 3 School of Pharmacy, East China University of Science and Technology, Shanghai, China; 4 Institute for Healthy China, Tsinghua University, Beijing, China; 5 Vanke School of Public Health, Tsinghua University, Beijing, China; 6 IDG/McGovern Institute for Brain Research, Tsinghua University, Beijing, China

**Keywords:** bacterial translocation, exertional heat stroke, gut microbiota, heat stress, intestinal barrier

## Abstract

**Purpose:**

The incidence of exertional heat stroke (EHS) has increased markedly in recent decades. Although intestinal barrier dysfunction and gut microbiota alterations are increasingly implicated in EHS pathophysiology, the respective contributions of heat exposure and physical exercise to these processes remain incompletely defined.

**Methods:**

Male C57BL/6 mice were assigned to Control (C), Exercise (E), Heat shock (H), or Exercise + Heat shock (HE) groups. Exercise and/or heat exposure were applied to induce exertional heat stress. Intestinal injury and permeability were assessed by histopathology and circulating D-lactate levels. Gut and blood microbial profiles were characterized using 16S rRNA gene sequencing, and associations between microbial signatures and intestinal injury markers were analyzed.

**Results:**

Both heat exposure and exercise induced intestinal injury and increased circulating D-lactate levels, with the most severe effects observed in the combined HE group. Heat exposure was associated with pronounced alterations in gut microbial diversity and community structure, whereas exercise was associated with increased microbial diversity and gut-associated microbial signatures detected in blood samples. Differential abundance analyses revealed distinct taxonomic profiles associated with heat, exercise, and their combination. Correlation analyses demonstrated significant associations between intestinal injury markers and circulating microbial profiles.

**Conclusion:**

These findings indicate that heat exposure and exercise exert distinct yet interacting associations with intestinal barrier integrity and microbial community distribution. Heat stress primarily disrupts gut microbial ecology and barrier function, whereas exercise is more closely associated with increased systemic detection of gut-derived microbial signatures. Together, these results highlight the gut microbiota–barrier axis as a key interface linking environmental and physiological stressors to systemic responses during exertional heat stress.

## Highlights

- Heat stress can disrupt the composition of the gut microbiota, and exercise can further exacerbate the leakage of these microorganisms into the bloodstream through the damaged intestinal barrier.
- Heat stress and exercise synergistically promote bacterial translocation and systemic inflammation, which may be the reason why exertional heat stroke can lead to multiple organ dysfunction.
- Interventions such as heat adaptation, probiotics, or barrier protectors are potential mitigation strategies.

## 1 Introduction

The gut microbiota represents a complex and dynamic microbial ecosystem that plays a fundamental role in maintaining intestinal barrier integrity and systemic physiological homeostasis. Through coordinated metabolic activities, immune modulation, and regulation of epithelial signaling, commensal microorganisms actively contribute to host defense and inflammatory balance (Hand et al., 2016; Thaiss et al., 2018). Disruption of this host–microbe symbiosis has been increasingly implicated in the pathogenesis of a wide range of disorders, including metabolic, inflammatory, and neurodegenerative diseases (Ni et al., 2017; Martel et al., 2022). A critical consequence of microbiota dysbiosis is the impairment of intestinal barrier function, which facilitates the translocation of gut-derived microbes or microbial products into the circulation, thereby converting a localized ecological disturbance into systemic inflammatory signaling (Manfredo Vieira et al., 2018).

Environmental stressors are increasingly recognized as potent modulators of gut microbial ecology. While chemical pollutants and dietary perturbations have been extensively studied, physical stressors—such as extreme heat—remain comparatively underexplored from a microbiological perspective. Experimental evidence indicates that heat exposure can disrupt epithelial tight junctions, alter mucus composition, and reshape gut microbial communities, leading to increased intestinal permeability (Hall et al., 2001; Sun et al., 2024). Several studies published in Frontiers in Microbiology and related journals have demonstrated that thermal stress induces marked shifts in gut microbiota composition and metabolic capacity, accompanied by compromised barrier function and heightened inflammatory responses (Xie et al., 2024; Liu et al., 2023). These findings support the concept that heat stress constitutes an ecological perturbation capable of destabilizing host–microbe equilibrium, analogous to classical environmental toxicants.

Exertional heat stroke (EHS), characterized by strenuous physical activity performed under high ambient temperatures, provides a clinically relevant model to investigate how combined thermal and mechanical stressors affect the gut microbiota–barrier axis. EHS is associated with rapid onset of systemic inflammation and multi-organ dysfunction, outcomes that cannot be fully explained by hyperthermia alone (Bouchama et al., 2022; Garcia et al., 2022; Casa et al., 2015). Accumulating evidence suggests that gastrointestinal barrier failure and gut-derived microbial leakage play central roles in disease progression. Elevated circulating endotoxin levels and detection of bacterial DNA or viable bacteria in the bloodstream of heat stroke patients support a “gut-origin” contribution to systemic injury (Bouchama et al., 1991; Ramírez et al., 2009; Armstrong et al., 2018; Ogden et al., 2020). However, the relative contributions of heat exposure and physical exertion to microbiota dysbiosis and microbial translocation remain poorly defined.

Physical exercise itself exerts bidirectional effects on the gut microbiota. Moderate and regular physical activity has been shown to enrich short-chain fatty acid–producing bacteria and enhance epithelial resilience, whereas intense or prolonged exercise may compromise splanchnic perfusion and increase intestinal permeability (Costa et al., 2017; Dmytriv et al., 2024; Cinca-Morros and Álvarez-Herms, 2024). When intense exercise is superimposed on heat-induced barrier vulnerability, the risk of microbial translocation may be substantially amplified. Recent studies have reported the presence of gut-associated bacterial DNA in the circulation following exertional heat stress, highlighting exercise as a potential accelerator of systemic microbial signaling (Henningsen et al., 2024). Nevertheless, direct experimental evidence disentangling the independent and synergistic effects of heat and exercise on both gut and blood microbiota remains limited.

In this study, we established a controlled mouse model combining exercise and heat stress to systematically investigate their respective and interactive effects on intestinal barrier integrity, gut microbiota composition, and microbial translocation into the bloodstream. By integrating histological assessment, circulating permeability biomarkers, and 16S rRNA gene sequencing of fecal and blood samples, we aimed to clarify how thermal and mechanical stressors jointly disrupt host–microbe homeostasis. We hypothesized that heat exposure primarily reshapes gut microbial ecology and compromises barrier integrity, whereas exercise amplifies the leakage of gut-derived microbial signals into the systemic circulation. Elucidating these mechanisms may provide microbiological insights into the rapid progression and systemic nature of exertional heat stroke and highlight the gut microbiota–barrier axis as a potential target for mitigating heat-related systemic injury under increasingly extreme environmental conditions.

## 2 Materials and methods

### 2.1 Establishment of heat stroke mouse model

C57BL/6 male mice aged 9–10 weeks were bred in the Animal Experiment Center of Tsinghua University under standard conditions. A total of 24 mice weighing (27.5 g ± 2.5 g) were randomly divided into four groups: the control group (C, 26 ± 0.5 °C), exercise group (E, 26 ± 0.5 °C), heat stress group (H, 42 ± 0.5 °C), and exertional heat stroke group (HE, 42 ± 0.5 °C), with 8 mice in each group to investigate the differences in weight, temperature, blood and fecal tests. The humidity in each group was (50 ± 5) %.

During the intervention, the mice in the HE group and the E group were made to exercise at 42 and 25 °C, respectively. The numerically controlled treadmill was set at a speed of 12 m/min in continuous running mode, with a 30-s rest every 10 min. Then, the rectal temperature of each experimental mouse was quickly measured and recorded using the body temperature detection probe of the vital sign detector. The state and body weight changes of the mice after high temperature and exercise were observed and recorded, and the change curve of the core temperature of the mice was drawn until the successful preparation criteria for the EHS model were achieved. When the mice in the HE group stopped exercising, the mice in the E group stopped simultaneously.

Successful establishment of the exertional heat model was defined based on established physiological criteria, including deep and labored breathing, cyanosis of the extremities, reduced posture with the abdomen close to the treadmill surface, and a rectal temperature reaching 42.0 °C.

At the endpoint of the experiment, mice were euthanized under deep anesthesia using isoflurane followed by cervical dislocation to ensure rapid and humane sacrifice. All samples were collected immediately after euthanasia as described below.

### 2.2 Histological analysis

Distal colon samples (~5 mm) were collected within 5 min following euthanasia and immediately fixed in 4% paraformaldehyde for 24 h. Then the sample was embedded in paraffin wax. The sample was sliced into 5 μm sections for hematoxylin and eosin (H&E) staining. Randomly selected slices from each group were observed with a pathologic slice scanner at 100 × magnification, and then photomicrographs were recorded. A pathology researcher who was blind to the grouping situation calculated the histological index (HI) according to the following criteria to assess the degree of histopathological changes (Supplementary Table S1).

### 2.3 Evaluation of intestinal barrier permeability

D-lactic acid is a metabolite primarily produced by bacterial fermentation in the gastrointestinal tract (Oh et al., 1979). Under physiological conditions, circulating D-lactate levels are typically low. When intestinal barrier integrity is compromised, D-lactate can translocate into the circulation (Turner, 2009). Circulating D-lactate levels have been reported to correlate with the severity of intestinal mucosal injury (Nielsen et al., 2011).

However, D-lactate is not exclusively microbiota-derived and may also originate from endogenous metabolic pathways, such as the methylglyoxal pathway (Zhou et al., 2022; Paoli et al., 2013). Therefore, D-lactate was interpreted as a supportive, rather than definitive, biomarker of intestinal permeability in this study.

Plasma enzyme-linked immunosorbent assay (ELISA) kits were used for the determination FABP-2 (SHUHUA BIOLOGY) concentrations and were performed according to the manufacturer's instructions.

### 2.4 Feces and blood collection

Fresh mouse feces from the cecum were collected into individual sterile EP tubes, quickly frozen on dry ice, and then transferred into an −80 °C cryogenic freezer for cryopreservation until DNA extraction.

Blood samples were obtained via cardiac puncture, placed in heparin or EDTA tubes, used immediately in point of care testing or spun at 3000 RCF for 5 min at 4 °C to separate plasma from the buffy coat, divided into aliquots and stored at −80 °C for later analysis. Whole blood samples were further subjected to DNA extraction for 16S rRNA gene sequencing as described below.

### 2.5 16S rRNA gene sequencing analysis

16S rRNA gene sequencing of mouse fecal samples was performed following the protocol of a recently published study Xu et al. (2023) with minor modifications. Briefly, after genomic DNA extraction, the V4 region of the 16S rRNA gene was amplified (515F/806R) and sequenced on an Illumina platform (2 × 250 bp). Raw reads were processed using QIIME2 (v2019.1) to generate amplicon sequence variants (ASVs), which were taxonomically classified against the Greengenes 13_8 (99%) database. Low-abundance features (singletons) were removed. Alpha and beta diversity indices were computed to evaluate community structure, and group differences were assessed using supervised and unsupervised multivariate analyses (e.g., linear discriminant analysis effect size, LEfSe). In addition to singleton removal, sequences were quality-filtered based on read length, Phred quality scores, and chimera removal using DADA2 within QIIME2. Samples with extremely low sequencing depth were excluded to ensure robustness of downstream analyses. Functional profiles were predicted using PICRUSt based on KEGG orthologs. Raw sequence data have been deposited in the NCBI Sequence Read Archive (SRA) under accession number PRJNA1233769.

To minimize the influence of background contamination in low-biomass blood samples, all blood and fecal samples were processed using identical DNA extraction, amplification, and sequencing pipelines. Negative controls and control-group blood samples consistently exhibited low microbial diversity and distinct community structures compared with exercise-exposed groups. Moreover, the selective enrichment of gut-associated taxa in blood samples from the Exercise and Exercise + Heat groups, together with significant correlations between intestinal injury markers and blood microbial diversity, supports a biological rather than artifactual origin of circulating microbial signals.

### 2.6 Statistical analyses

Data were presented as the mean ± standard error of the mean or median and interquartile range. For non-normally distributed data, the Kruskal-Wallis test was used for analysis; for normally distributed data, one-way ANOVA analysis was adopted, followed by Tukey's test. All statistical analyses and graph plotting were performed using Graph Pad Prism (version 9.0.0), and a *P* < 0.05 was considered statistically significant.

## 3 Results

### 3.1 Intestinal injury and increased permeability under exertional heat stress

**Figure 1:**
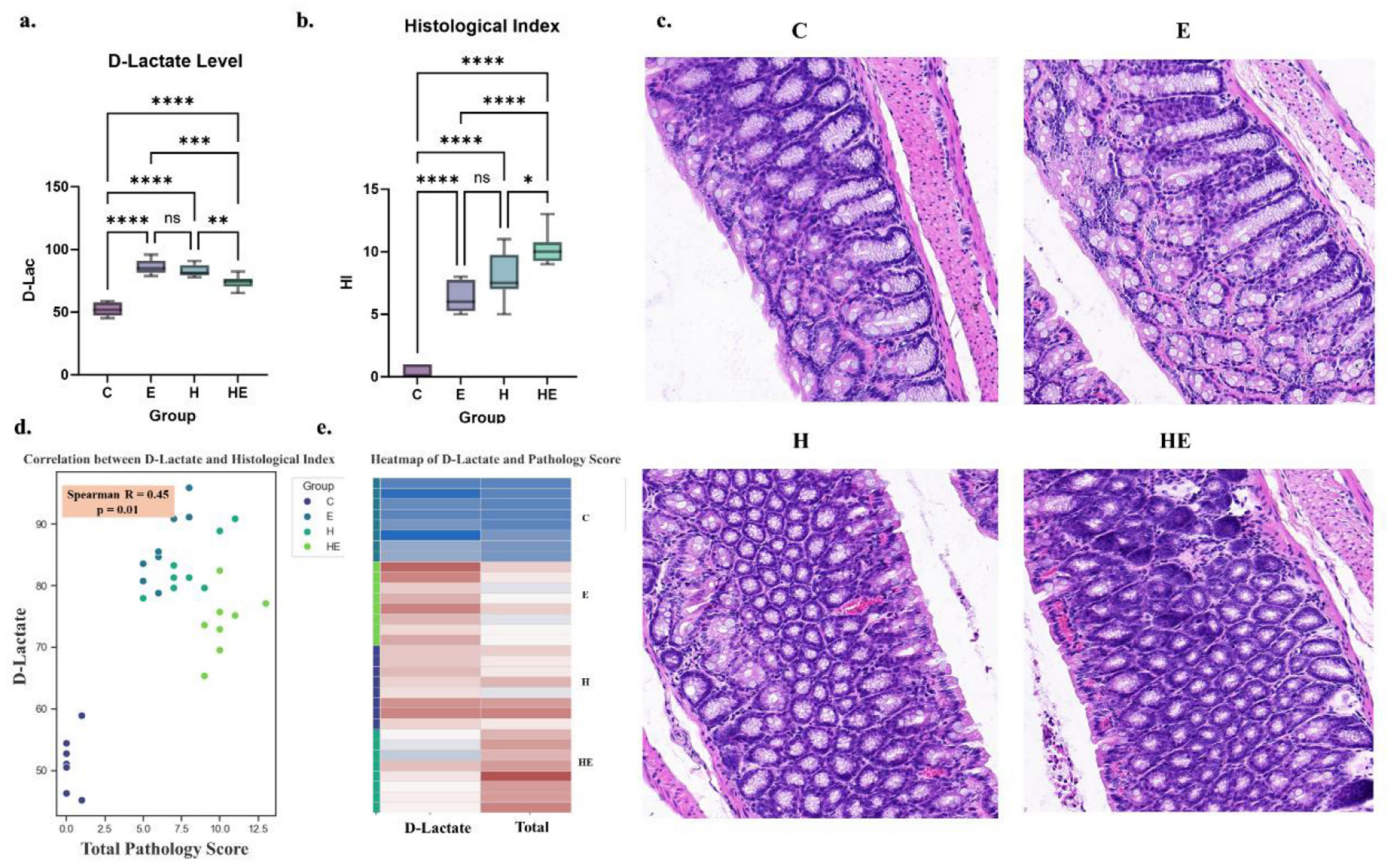
Analysis of intestinal permeability and gut injury. **(a)** Blood D-Lactate levels in each group. **(b)** Total pathology scores of intestinal tissue for each group. **(c)** HE staining results of intestinal tissues from four groups of mice. **(d)** Scatter plot showing the correlation between total pathology score and blood D-Lactate levels. **(e)** Heatmap of D-Lactate levels and total pathology scores for individual mice. Asterisks indicate significant differences between groups as determined by *post-hoc* tests: *p < 0.05, **p < 0.01, ***p < 0.001, ****p < 0.0001. ns, not significant.

Blood D-lactate concentrations and histopathological assessment were used to evaluate intestinal barrier integrity following heat and/or exercise exposure. Significant differences in circulating D-lactate levels were observed among groups. Compared with the Control (C) group, the Exercise (E), Heat shock (H), and Exercise + Heat shock (HE) groups all exhibited significantly elevated D-lactate levels (*p* < 0.0001; Figure 1a). Similarly, total intestinal pathology scores were significantly higher in the E, H, and HE groups than in the C group (Figures 1b, c). Importantly, the HE group exhibited the most severe intestinal injury, with pathology scores significantly exceeding those of both the E (*p* < 0.05) and H (*p* < 0.0001) groups.

Spearman correlation analysis demonstrated a moderate positive correlation between circulating D-lactate levels and total intestinal pathology scores (*R* = 0.45, *p* = 0.010; Figure 1d), which was further visualized at the individual level using a heatmap (Figure 1e). Together, these results indicate that combined heat and exercise stress induces pronounced intestinal injury and increases intestinal permeability, a physiological state that may facilitate the passage of luminal microbial components.

### 3.2 Alterations in gut and blood microbiota profiles in response to heat and exercise

#### 3.2.1 Alpha-diversity responses of fecal and blood microbiota

**Figure 2:**
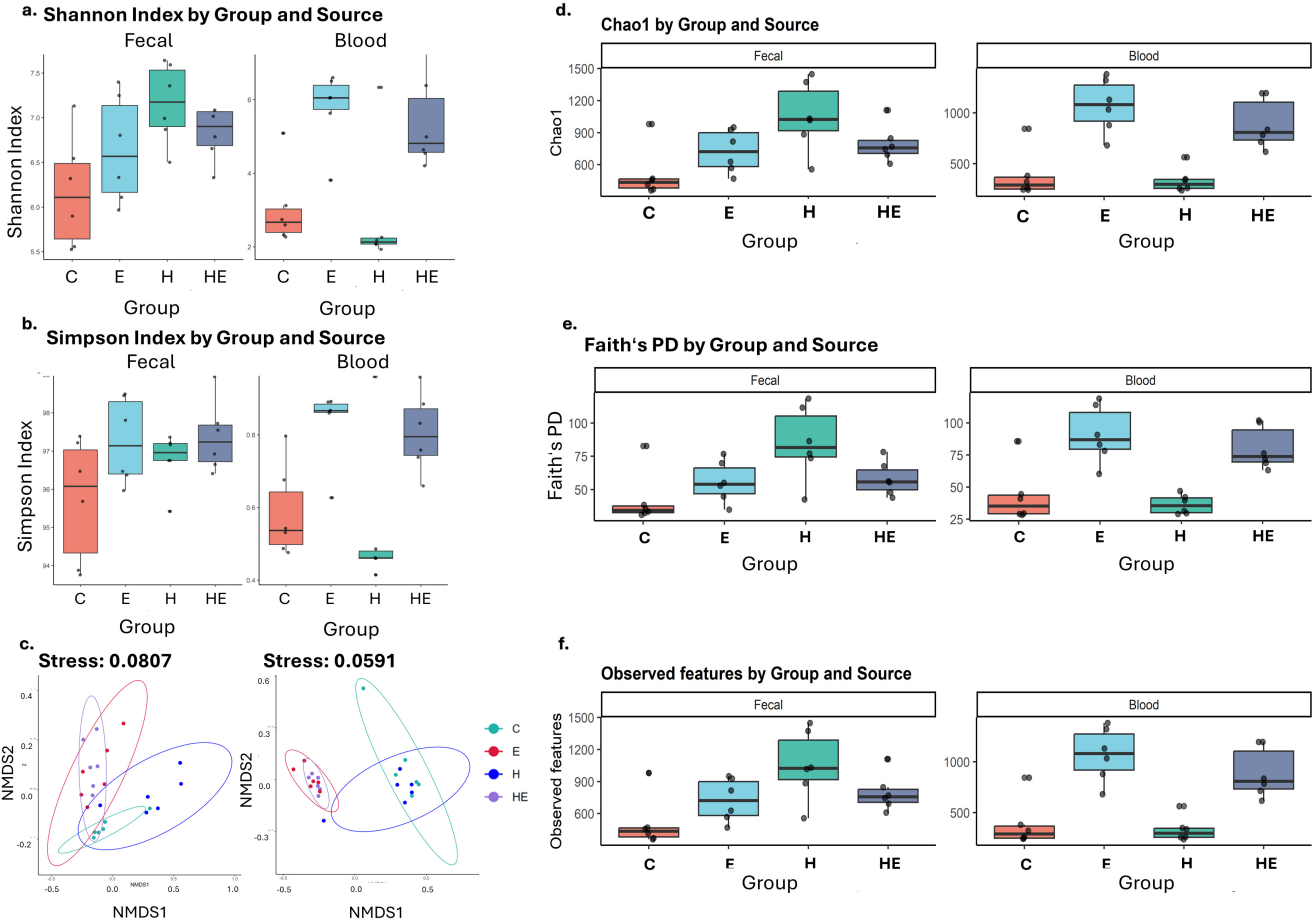
Alterations in alpha and beta diversity of fecal and blood microbiota in response to heat and exercise. **(a)** Between-group differences in Shannon entropy for fecal and blood microbiota. **(b)** Between-group differences in the Simpson index for fecal and blood microbiota. **(c)** Non-metric multidimensional scaling (NMDS) plots based on unweighted UniFrac distances for fecal microbiota (stress = 0.0807) and blood microbiota (stress = 0.0591). **(d)** Between-group differences in the Chao1 index for fecal and blood microbiota. **(e)** Between-group differences in the Faith's PD index for fecal and blood microbiota. **(f)** Between-group differences in the Observed Features index for fecal and blood microbiota.

Alpha-diversity indices were calculated to assess within-sample microbial richness and evenness. In fecal samples, Shannon and Simpson indices were significantly higher in the Heat shock (H) and Exercise + Heat shock (HE) groups than in the Control (C) group (Figures 2a, b). Consistently, richness-related indices, including Chao1, Faith's phylogenetic diversity, and Observed Features, were also highest in heat-exposed groups (Figures 2d–f), indicating that heat stress was associated with marked alterations in gut microbial diversity.

In contrast, blood samples from exercise-exposed mice (E and HE groups) exhibited significantly higher alpha-diversity and richness indices than those from the C and H groups (Figures 2a, b, d–f). These findings indicate that exercise exposure was associated with increased microbial diversity detected in the circulation, whereas heat exposure alone exerted a more limited effect on blood-associated microbial diversity.

#### 3.2.2 Beta-diversity shifts in fecal and blood microbiota

Non-metric multidimensional scaling (NMDS) analysis based on unweighted UniFrac distances was used to evaluate between-sample community dissimilarity. In fecal samples, the H group formed a distinct cluster separated from the C, E, and HE groups along the NMDS1 axis (stress = 0.0807; Figure 2c), indicating that heat exposure was strongly associated with shifts in gut microbial community structure.

In blood samples, microbial profiles from the C group clustered tightly, reflecting low inter-individual variability. In contrast, samples from the E and HE groups exhibited greater dispersion and clustered separately from the C and H groups (stress = 0.0591; Figure 2c), suggesting that exercise was the dominant factor associated with changes in blood-associated microbial community structure.

#### 3.2.3 Taxonomic composition of fecal and blood microbiota

**Figure 3:**
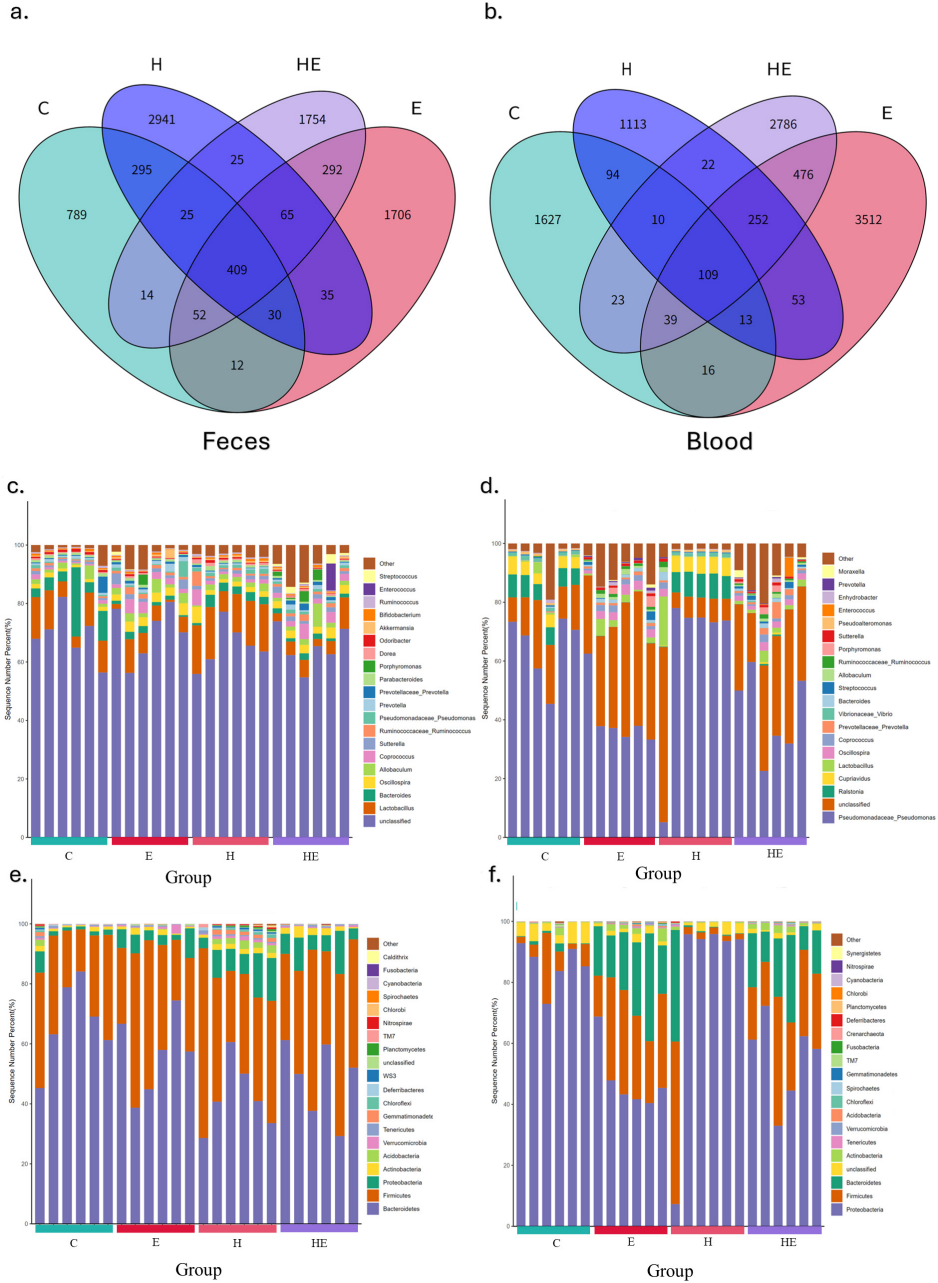
Taxonomic composition and feature distribution. **(a,b)** Venn diagrams showing the number of shared and unique features in fecal and blood samples across the different groups, respectively. **(c,d)** Stacked bar plots of relative abundance at the genus level for fecal and blood microbiota, respectively. **(e,f)** Stacked barplots of relative abundance at the phylum level for fecal and blood microbiota, respectively.

Venn diagram analysis revealed that the H group harbored the largest number of unique amplicon sequence variants (ASVs) in fecal samples (2,941), whereas the E group exhibited the highest number of unique ASVs in blood samples (3,512; Figures 3a, b). At the genus level, fecal samples from the C and E groups were relatively enriched in Prevotella, whereas Bacteroides showed higher relative abundance in the H and HE groups. Lactobacillus abundance was reduced in heat-exposed groups (H and HE) compared with the C group (Figure 3c).

In blood samples, genera including *Prevotellaceae_Prevotella, Ralstonia*, and *Cupriavidus* were more abundant in the C and H groups than in the exercise-exposed groups (Figure 3d). These observations indicate that gut microbial composition was more strongly associated with heat exposure, whereas blood-associated microbial profiles were more closely related to exercise status.

At the phylum level in fecal samples, Bacteroidetes and Firmicutes were the predominant taxa across all groups. Compared with the C and CE groups, H and HE groups exhibited a relative decrease in the abundance of Bacteroidetes, while the proportions of Firmicutes and Proteobacteria showed a corresponding increase in several samples (Figure 3e).

In blood samples, the microbial composition at the phylum level was characterized by a high dominance of Proteobacteria in the C and H groups. However, in the E and HE groups, the relative abundance of Proteobacteria was markedly lower, accompanied by a notable expansion of Firmicutes and Bacteroidetes (Figure 3f).

#### 3.2.4 Differentially abundant taxa identified by LEfSe and volcano plot analyses

**Figure 4:**
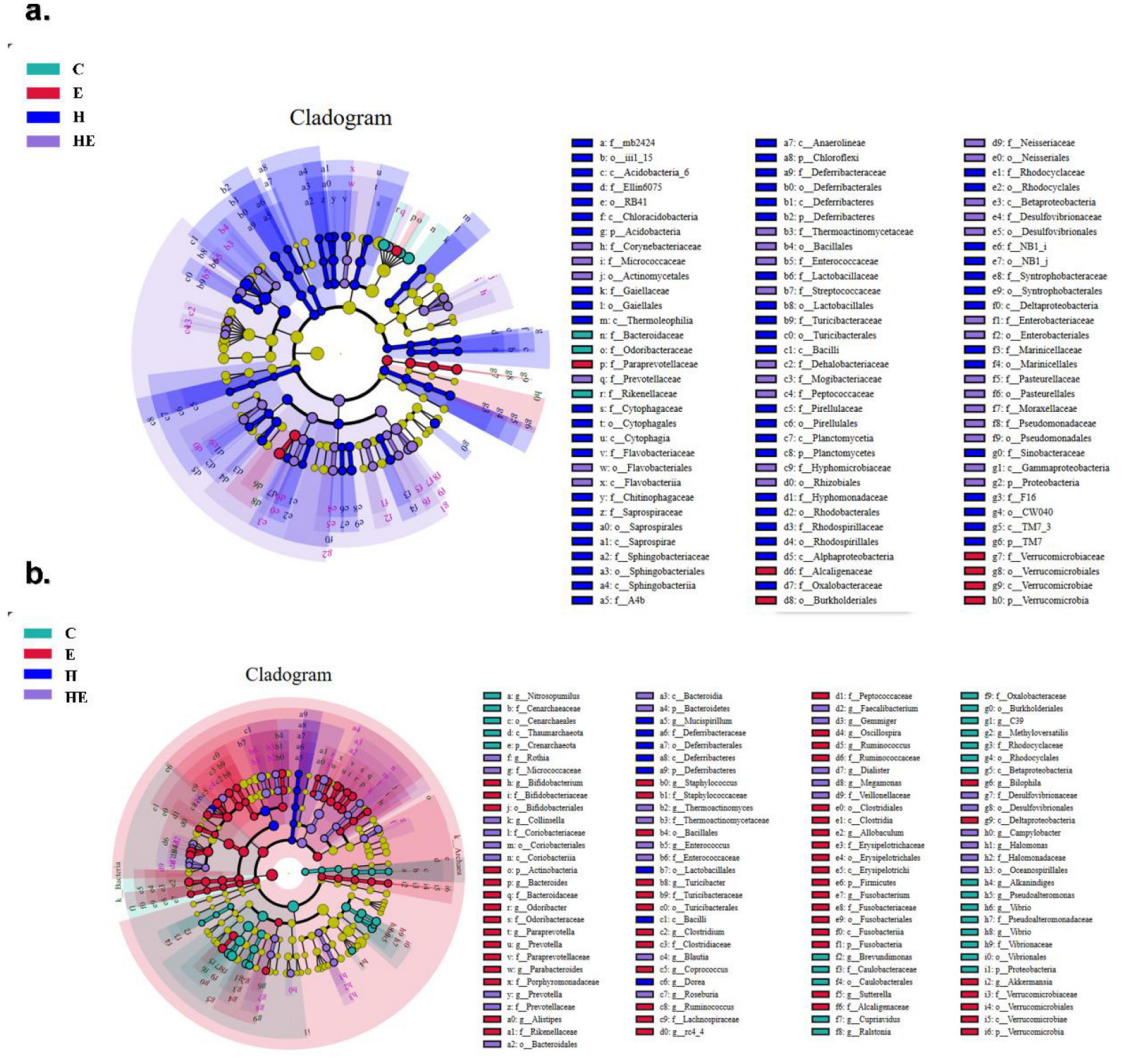
LEfSe cladogram of blood and fecal microbiota across groups. **(a)** LEfSe cladogram for blood samples from different groups. **(b)** LEfSe cladogram for fecal samples from different groups. The colored nodes represent microbial taxa that are significantly enriched in the corresponding groups, while the yellow nodes indicate taxa that show no significant differences across the groups.

LEfSe analysis was performed to identify microbial taxa differentially associated with heat and/or exercise exposure in fecal and blood samples (Figure 4). In fecal microbiota, the H group was characterized by increased relative abundances of *Proteobacteria* and *Desulfovibrionaceae* and decreased abundances of *Lachnospiraceae* and *Ruminococcaceae* compared with the C group. In contrast, the E group exhibited enrichment of multiple taxa belonging to *Lachnospiraceae, Ruminococcaceae, Lactobacillus*, and *Bacteroides*.

In blood samples, the H group showed higher relative abundances of *Enterobacteriaceae, Streptococcaceae*, and *Clostridium_sensu_stricto*, whereas exercise-exposed groups were characterized by increased detection of several gut-associated taxa. Notably, the Exercise + Heat shock (HE) group displayed a microbial profile distinct from either stressor alone.

#### 3.2.5 Identify differentially abundant taxa across treatment groups

**Figure 5:**
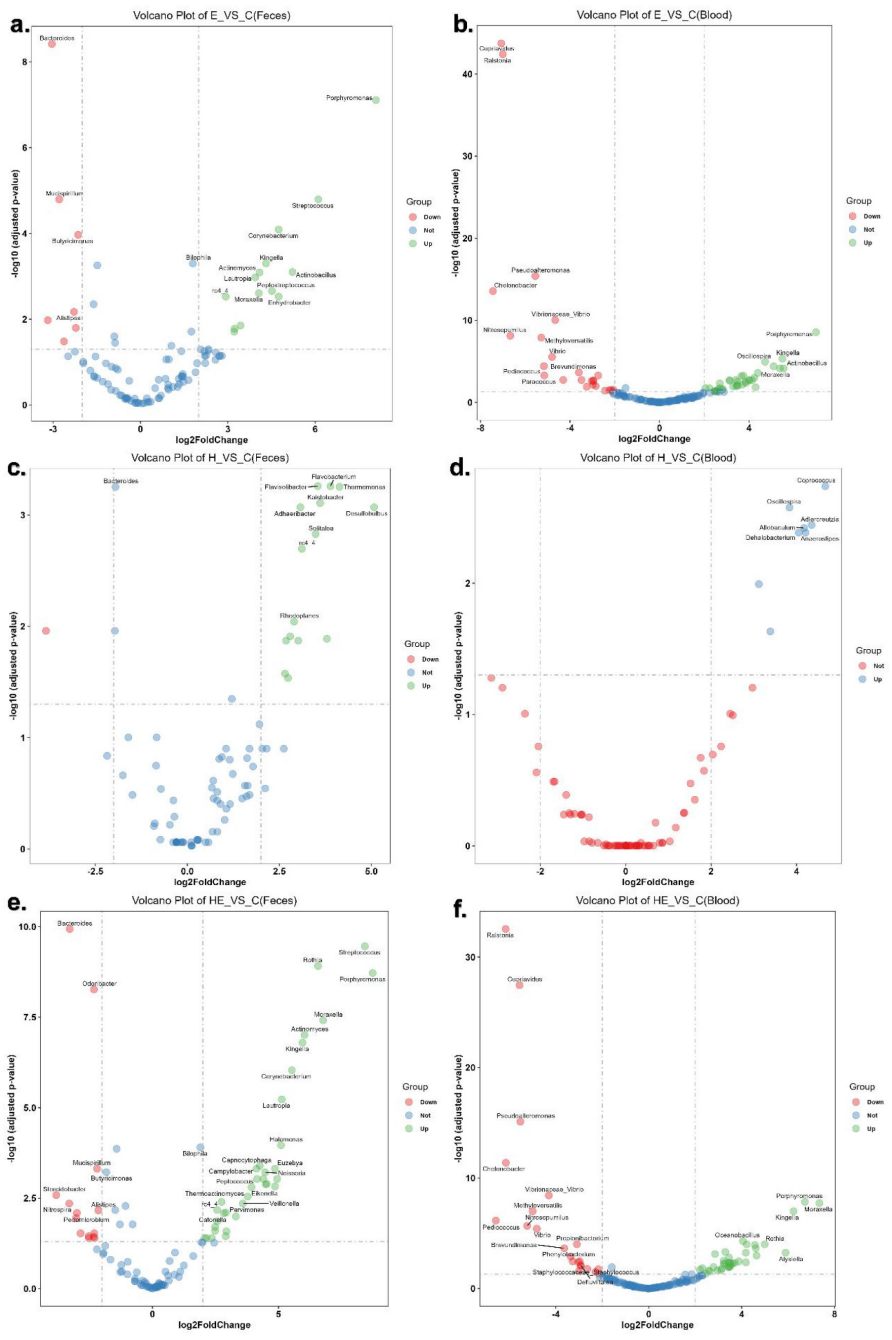
Volcano plots of differentially abundant taxa. **(a,b)** Significantly altered taxa in the Exercise (E) group relative to the Control (C) group for fecal and blood samples, respectively. **(c,d)** Significantly altered taxa in the Heat shock (H) group relative to the Control (C) group for fecal and blood samples, respectively. **(e,f)** Significantly altered taxa in the Exercise + Heat shock (HE) group relative to the Control (C) group for fecal and blood samples, respectively.

To further resolve group-specific differences, differential abundance analysis was visualized using volcano plots (Figure 5). In fecal samples, the HE group showed significant enrichment of *Streptococcus, Rothia, Porphyromonas, Moraxella*, and *Actinomyces*, accompanied by significant depletion of *Bacteroides, Odoribacter, Mucispirillum, Steroidobacter*, and *Nitrospira* relative to the Control group (Figure 5e; Table 1). In blood samples, the HE group was characterized by increased relative abundances of *Porphyromonas, Moraxella, Kingella, Oceanobacillus*, and *Rothia*, while *Ralstonia, Cupriavidus, Pseudoalteromonas, Chelonobacter*, and *Vibrionaceae_Vibrio* were significantly decreased compared with the Control group (Figure 5f; Table 1).

**Table 1:** Top 5 differentially abundant genera in each comparison group relative to the control.

Sample type	Group (vs. control)	Significantly decreased	Significantly increased
Feces	E	*Bacteroides, Mucispirillum, Butyricimonas, Alistipes, Nitrospira*	*Porphyromonas, Streptococcus, Corynebacterium, Kingella, Actinobacillus*
	H	*Blautia*	*Flavobacterium, Flavisolibacter, Thermomonas, Kaistobacter, Adhaeribacter*
	HE	*Bacteroides, Odoribacter, Mucispirillum, Steroidobacter, Nitrospira*	*Streptococcus, Rothia, Porphyromonas, Moraxella, Actinomyces*
Blood	E	*Cupriavidus, Ralstonia, Pseudoalteromonas, Chelonobacter, Vibrionaceae_Vibrio*	*Porphyromonas, Kingella, Oscillospira, Coprococcus, Actinobacillus*
	H	None	*Coprococcus, Oscillospira, Adlercreutzia, Allobaculum, Dehalobacterium*
	HE	*Ralstonia, Cupriavidus, Pseudoalteromonas, Chelonobacter, Vibrionaceae_Vibrio*	*Porphyromonas, Moraxella, Kingella, Oceanobacillus, Rothia*

### 3.3 Circulating microbial signatures and their association with intestinal injury

**Figure 6:**
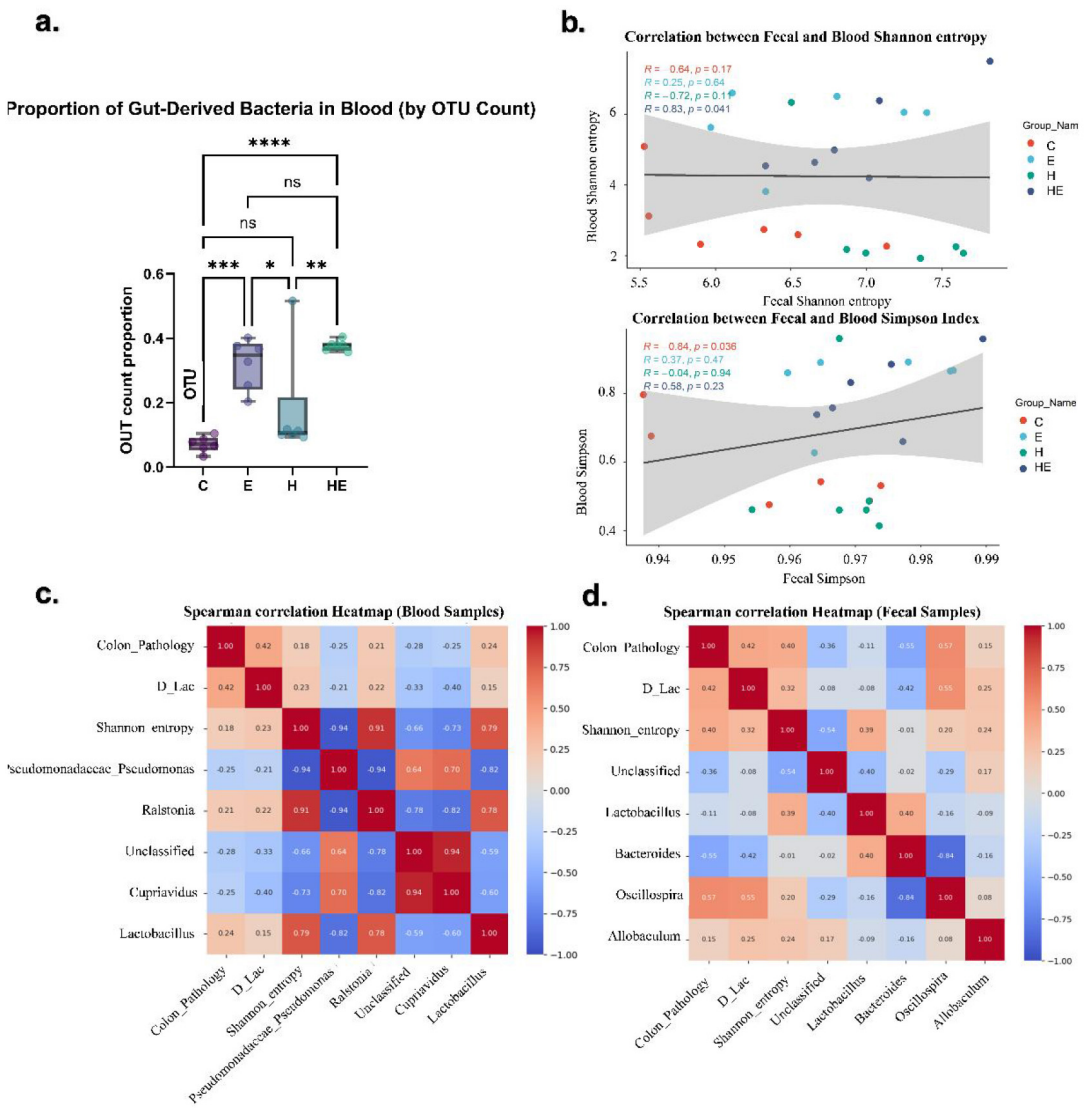
Microbial translocation and correlation analysis. **(a)** Proportion of gut-derived bacteria in the blood for each group. **(b)** Correlation analysis of alpha diversity indices (Shannon and Simpson) between fecal and blood samples. **(c,d)** Spearman correlation heatmaps of all analytical variables for blood and fecal samples, respectively. *P < 0.05, **P < 0.01, ***P < 0.001, and ****P < 0.0001.

The proportion of gut-associated microbial signatures detected in blood samples differed significantly among groups. This proportion was significantly higher in the E (32.16%) and HE (37.73%) groups than in the C (7.12%) and H (17.33%) groups (*p* < 0.001; Figure 6a). In the HE group, fecal and blood Shannon diversity indices were positively correlated (*R* = 0.83, *p* = 0.041; Figure 6b).

Correlation analyses further revealed significant associations between microbial taxa and host injury markers. Total intestinal pathology scores were positively correlated with *Oscillospira* abundance in blood (*R* = 0.57, *p* = 0.004) and negatively correlated with blood *Bacteroides* abundance (*R* = −0.55, *p* = 0.006). Circulating D-lactate levels were positively correlated with *Oscillospira* abundance in fecal samples (*R* = 0.55, *p* = 0.005). In addition, blood Shannon diversity showed a strong negative correlation with *Pseudomonadaceae_Pseudomonas* (*R* = −0.94, *p* < 0.001) and a positive correlation with *Lactobacillus* abundance (*R* = 0.79, *p* < 0.001; Figures 6c, d). 3.3. Exacerbate microbial translocation and link it to gut injury markers under exercise stress.

## 4 Discussion

In the present study, we investigated the independent and combined effects of heat exposure and exercise on intestinal barrier integrity, gut microbiota composition, and circulating microbial signatures using a controlled mouse model. Our results demonstrate that heat stress is strongly associated with intestinal barrier injury and marked alterations in gut microbial community structure, whereas exercise is more closely associated with changes in microbial diversity and composition detected in the circulation. These findings support a conceptual framework in which thermal and mechanical stressors exert complementary, rather than redundant, effects on the gut microbiota–barrier axis.

Heat exposure alone induced substantial intestinal injury, as evidenced by increased histopathological scores and elevated circulating D-lactate levels. These observations are consistent with established evidence that hyperthermia compromises epithelial integrity through mechanisms including reduced splanchnic perfusion, oxidative stress, and tight junction disruption (Hall et al., 2001; Sun et al., 2024). Importantly, concomitant shifts in gut microbial diversity and taxonomic composition were observed in heat-exposed mice, indicating that thermal stress acts as a potent ecological perturbation to the intestinal microbiota. Similar heat-associated microbiota alterations have been reported in rodent models and humans, including studies published in Frontiers in Microbiology, which demonstrated that heat stress or heat acclimation reshapes gut microbial communities and is linked to intestinal and systemic outcomes (Liu et al., 2023; Li et al., 2024).

While heat exposure predominantly affected the gut compartment, exercise exerted a distinct influence on circulating microbial profiles. Exercise-exposed groups exhibited increased microbial diversity and a higher proportion of gut-associated microbial signatures in blood samples, even in the absence of the most severe histological injury. Previous studies have shown that intense or prolonged exercise can transiently increase intestinal permeability by reducing mesenteric blood flow and inducing epithelial stress, thereby facilitating the systemic appearance of microbial components (Costa et al., 2017; Dmytriv et al., 2024). Recent human data further support this interpretation, demonstrating increased detection of bacterial DNA in plasma following exertional heat stress (Henningsen et al., 2024). Together with our findings, these observations suggest that exercise may amplify systemic exposure to microbial signals once intestinal barrier integrity is compromised.

It is noteworthy that our findings reveal a specific pattern of microbial dysbiosis induced by the combination of exercise and heat stress. While the unweighted UniFrac analysis indicates a conserved overall taxonomic composition (presence/absence), the taxonomic profiling (Figure 5) demonstrates profound shifts in relative abundance. This suggests that the exertional heat stroke model does not necessarily eliminate existing species or introduce novel ones, but rather severely disrupts the ecological equilibrium of the gut. This proportional imbalance—marked by the expansion of opportunistic populations at the expense of beneficial commensals—appears to be a critical driver of the systemic microbial translocation observed in the HE group.

Notably, combined heat and exercise exposure resulted in a microbial configuration distinct from either stressor alone. LEfSe and differential abundance analyses revealed a redistribution of multiple taxa across intestinal and circulatory compartments in the HE group, rather than a simple additive effect of heat- or exercise-associated profiles. This pattern suggests that combined stress induces a unique microbial state characterized by altered community structure and compartmentalization. Similar non-additive microbiota responses to combined environmental and physiological stressors have been reported in recent microbiome studies, underscoring the dynamic and context-dependent nature of host–microbe interactions under extreme conditions (Xie et al., 2024).

From a microbiological perspective, the detection of microbial DNA in blood samples has important implications for understanding systemic responses to exertional heat stress. Although blood has traditionally been considered sterile, accumulating evidence indicates that circulating microbial signatures can emerge under pathological or extreme physiological conditions and reflect disrupted barrier function and altered host–microbe interactions (Manfredo Vieira et al., 2018). While 16S rRNA gene sequencing does not distinguish viable bacteria from bacterial fragments, prior work has demonstrated that microbial components alone are sufficient to activate innate immune pathways and systemic inflammation (Bouchama et al., 1991; Leon and Helwig, 2010; Luo et al., 2023). Therefore, the associations observed here between intestinal injury markers and circulating microbial profiles support a potential contribution of gut-derived microbial signals. Notably, the increased alpha diversity observed in blood samples from exercise-exposed groups provides quantitative support for the presence of a broader range of microbial DNA, consistent with enhanced translocation of gut-derived microbial components into the circulation. However, when interpreting biomarkers of intestinal barrier disruption, certain limitations should be considered. While D-lactate provides supportive evidence of barrier disruption, it is not exclusively derived from microbial sources and should be interpreted in conjunction with other indicators. Additionally, D-lactate may also arise from endogenous metabolic pathways, which should be considered when interpreting its levels (Zhou et al., 2022; Paoli et al., 2013).

Several limitations of this study should be acknowledged. First, while 16S rRNA gene sequencing enabled the detection of microbial DNA in blood, this approach does not distinguish between viable bacteria and bacterial fragments. Nevertheless, previous studies have demonstrated that circulating bacterial DNA alone is sufficient to trigger inflammatory signaling pathways (Manfredo Vieira et al., 2018). Second, the use of a mouse model may not fully recapitulate the complexity of human EHS; however, the controlled experimental design allowed us to disentangle the relative contributions of heat and exercise, which is difficult to achieve in clinical settings. Third, we did not perform metagenomic or metabolomic profiling; thus, we lack direct evidence of microbial metabolic changes (e.g., SCFAs, endotoxins) influencing host responses. Prior work suggests that metabolites like SCFAs may modulate intestinal integrity and systemic inflammation (Sun et al., 2024). Future studies incorporating metagenomic or culture-based approaches, as well as targeted interventions such as probiotics or barrier-protective strategies, may further clarify causal relationships and translational relevance. In addition, D-lactate is not exclusively derived from microbial sources and may also reflect endogenous metabolic processes, which should be considered when interpreting it as a marker of intestinal permeability.

In the present study, taxonomic profiling was performed down to the species level to capture a detailed map of the microbial response to exertional heat stress. However, it is important to note that 16S rRNA V4 region sequencing often lacks the resolution required to distinguish between closely related species within the same genus. Consequently, while significant shifts were observed in certain well-characterized species such as *Akkermansia muciniphila*, a portion of the sequences could only be confidently assigned at the genus level. Our results highlight the dramatic restructuring of the gut microbiota—characterized more by proportional shifts in dominant genera than by the introduction of novel species—though further metagenomic explorations are warranted to achieve a more definitive species-level resolution.

Overall, our findings indicate that heat stress primarily disrupts intestinal barrier integrity and gut microbial ecology, whereas exercise is more closely associated with increased systemic detection of microbial signatures. By demonstrating distinct yet interacting associations of heat and exercise with gut and blood microbiota, this study highlights the gut microbiota–barrier axis as a central component linking environmental and physiological stressors to systemic responses during exertional heat stress.

## 5 Conclusion

In conclusion, our findings indicate that heat stress disrupts gut microbial ecology and intestinal barrier integrity, while exercise performed under heat stress is associated with enhanced systemic detection of gut-derived microbial signatures. Rather than reversing heat-associated microbial alterations, exercise appears to reshape the gut microbiota toward a distinct microbial configuration under combined stress conditions.

Together, these observations highlight the gut microbiota–barrier axis as a critical interface linking environmental and physiological stressors to systemic microbial signaling. By implicating microbial redistribution and barrier dysfunction as interacting processes during exertional heat stress, this study provides a microbiological framework for understanding how combined heat and exercise may contribute to amplified systemic responses.

## Data Availability

The datasets presented in this study can be found in online repositories. The names of the repository/repositories and accession number(s) can be found in the article/Supplementary material.
