# Supplementary material for: Combined heat and exercise stress disrupt gut microbiota and promote microbial translocation
Source: Front Microbiol. 2026 May 25;17:1779295. doi: 10.3389/fmicb.2026.1779295 (PMC13243434; doi:10.3389/fmicb.2026.1779295)
Supplement: Supplementary file 1 [file Table_1.docx]

Table S1 Standard score for colonic histopathology

| Scoring Item | Scoring Grade | Description |
| --- | --- | --- |
| Inflammatory cells infiltration | 0 | Inflammatory cells scattered in lamina propria mucosa |
|  | 1 | Increase of inflammatory cells in lamina propria mucosa |
|  | 2 | Inflammatory cells aggregate and invade the submucosa |
|  | 3 | Inflammatory cell transmural |
| Mucosal injury | 0 | The mucosa is regular and normal in shape. |
|  | 1 | Dispersed epithelial injury |
|  | 2 | Surface mucosal erosion or focal ulcer |
|  | 3 | Mucosal damage extends to the deep intestinal wall |
| Glandular fossa injury | 0 | The shape of glandular fossa is normal |
|  | 1 | 1/3 area injury in base |
|  | 2 | 2/3 area injury in base |
|  | 3 | Only superficial epithelial cells are intact |
|  | 4 | The glandular fossa disappears completely |
| The degree of regeneration and repair of mucosal epithelium | 0 | Complete repair or normal tissue |
|  | 1 | Nearly complete regenerative repair |
|  | 2 | Mucosal epithelium regenerate, but crypt is absent |
|  | 3 | Only the superficial epithelial cells are not damaged |
|  | 4 | Non-regenerative repair of mucosal epithelium |
